# Ischemia Reperfusion Injury Produces, and Ischemic Preconditioning Prevents, Rat Cardiac Fibroblast Differentiation: Role of K_ATP_ Channels

**DOI:** 10.3390/jcdd6020022

**Published:** 2019-06-04

**Authors:** Kartika R. Pertiwi, Rachael M. Hillman, Coralie A. Scott, Emily Lisa Chilton

**Affiliations:** 1 Department of Biology Education, Faculty of Mathematics and Natural Science, Yogyakarta State University, Yogyakarta 55281, Indonesia; kartika.pertiwi@uny.ac.id; 2 Physiology and Pharmacology, College of Public, Medical and Veterinary Sciences, James Cook University, 4814 Townsville, Queensland, Australia; Rachael.kaye@my.jcu.edu.au (R.M.H.); Coralie.scott@jcu.edu.au (C.A.S.)

**Keywords:** plasmalemmal K_ATP_ channels, mitochondrial K_ATP_ channels, myofibroblasts, α-smooth muscle actin, fibrosis

## Abstract

Ischemic preconditioning (IPC) and activation of ATP-sensitive potassium channels (K_ATP_) protect cardiac myocytes from ischemia reperfusion (IR) injury. We investigated the influence of IR injury, IPC and K_ATP_ in isolated rat cardiac fibroblasts. Hearts were removed under isoflurane anesthesia. IR was simulated in vitro by application and removal of paraffin oil over pelleted cells. Ischemia (30, 60 and 120 min) followed by 60 min reperfusion resulted in significant differentiation of fibroblasts into myofibroblasts in culture (mean % fibroblasts ± SEM in IR vs. time control: 12 ± 1% vs. 63 ± 2%, 30 min ischemia; 15 ± 3% vs. 71 ± 4%, 60 min ischemia; 8 ± 1% vs. 55 ± 2%, 120 min ischemia). IPC (15 min ischemia, 30 min reperfusion) significantly attenuated IR-induced fibroblast differentiation (52 ± 3%) compared to 60 min IR. IPC was mimicked by opening K_ATP_ with pinacidil (50 μM; 43 ± 6%) and by selectively opening mitochondrial K_ATP_ (mK_ATP_) with diazoxide (100 μM; 53 ± 3%). Furthermore, IPC was attenuated by inhibiting K_ATP_ with glibenclamide (10 μM; 23 ± 5%) and by selectively blocking mK_ATP_ with 5-hydroxydecanoate (100 μM; 22 ± 9%). These results suggest that (a) IR injury evoked cardiac fibroblast to myofibroblast differentiation, (b) IPC attenuated IR-induced fibroblast differentiation, (c) K_ATP_ were involved in IPC and (d) this protection involved selective activation of mK_ATP_.

## 1. Introduction

Cardiac ischemia reperfusion (IR) injury describes the damage caused by reduced coronary blood flow, causing depletion of ATP, reduced partial pressure of oxygen (PO_2_), acidosis, and build-up of toxins [1]. Reperfusion leads to further damage through generation of oxygen free radicals and a proton gradient across both the sarcolemma and the inner mitochondrial membrane [2,3].

Ischemic preconditioning (IPC) was first described by Murry et al. [4] and is classically defined as one or more cycles of brief IR injury, which protect the heart against a subsequent prolonged ischemic insult. The mechanisms responsible for generating this protection are complex and debated; however, the activation of ATP-sensitive potassium (K_ATP_) channels has been strongly implicated [5]. K_ATP_ channels were first described on the sarcolemmal membrane [6] of cardiac myocytes and were later discovered on the inner mitochondrial membrane [7]. Mitochondrial (mK_ATP_) and sarcolemmal (sK_ATP_) K_ATP_ channels are postulated to evoke cardioprotection by hyperpolarizing the sarcolemma and depolarizing the inner mitochondrial membrane [8] and/or by activation of intracellular signaling cascades [9]. Activation of mK_ATP_ channels attenuates mitochondrial Ca^2+^ entry via the Ca^2+^ uniporter [10,11] while activation of sK_ATP_ channels decreases voltage-gated Ca^2+^ current and action potential duration [12,13]. These actions reduce Ca^2+^ entry into the sarcoplasm and the mitochondria, thereby preventing Ca^2+^ overload-induced injury and death [8,12].

While both IR and IPC have been extensively studied in cardiac myocytes, their actions in non-myocyte cells of the heart have been poorly studied. The primary non-myocyte cell type of the heart is the cardiac fibroblast. Despite their small size, cardiac fibroblasts account for approximately two thirds of the total cell count in the heart [14,15]. Cardiac fibroblasts are important modulators of structure and function [16] in the healthy heart.

Following myocardial infarction, cardiac fibroblasts differentiate into the α-smooth muscle actin (α-SMA) expressing form, the myofibroblast [17,18]. Myofibroblasts are distinguished from fibroblasts by their role in the development of post-infarction pathologies. Myofibroblasts may deposit excessive extracellular matrix proteins within the infarcted heart. This leads to cardiac fibrosis and subsequent development of electrical and mechanical disturbances of the heart [17,19].

We investigated whether IR injury evoked rat cardiac fibroblast to myofibroblast differentiation and subsequently determined whether IPC had a protective influence on IR injury-induced fibroblast differentiation, and whether plasmalemmal K_ATP_ (pK_ATP_) channels and/or mK_ATP_ channels were involved in this protection.

## 2. Materials and Methods

### 2.1. Ethical Approval

All procedures were performed with ethical approval A1428 (2009, 2011) from the James Cook University Human and Animal Experimentation Ethics Committee and in compliance with the Australian code for the care and use of animals for scientific purposes, 8th edition (2013).

### 2.2. Ventricular Fibroblast Isolation

Isolation procedures were described previously [20]. Female Sprague Dawley rats (250–300 g) were injected with 0.15 mL of 1,000 IU heparin and anesthetized using isoflurane gas (5% induction, 1.5–2% maintenance) to the surgical plane. Isolated rat hearts were Langendorff-perfused at 37 °C and 8 mL min^-1^ with (a) Tyrode’s solution containing (mM): 140 NaCl, 5.4 KCl, 1 Na_2_HPO_4_, 5 HEPES, 1 MgCl_2_, 1 CaCl_2_ and 10 glucose for 5 min; (b) Ca^2+^-free Tyrode’s solution for 5 min and (c) Tyrode’s solution containing 0.04 mM CaCl_2_, 0.004 mg mL^−1^ protease (type XIV, Sigma, Castle Hill, NSW, Australia) and 0.04 mg mL^−1^ collagenase (type II, Worthington, Lakewood, NJ, USA) for ~12 min. Cells from the ventricles and septum were then dissociated in Tyrode’s solution containing 0.1 mM CaCl_2_, 0.1 mg mL^−1^ protease, 1 mg mL^−1^ collagenase and 5 mg mL^−1^ bovine serum albumin (BSA, Sigma) and shaken at 34 °C for ~40 min to finish dissociating cells. Following final digestion, cells were maintained under sterile conditions.

### 2.3. Characteristics of pH, PO_2_ and PCO_2_ During Ischemia and Reperfusion

Dissociated cells were centrifuged and resuspended in Dulbecco’s modified Eagle’s medium (DMEM, Gibco, Thermo Fisher Scientific, Waltham, MA, USA) containing penicillin/streptomycin (100 μg mL^-1^), amphotericin B (0.25 μg mL^-1^) and gentamicin (100 μg mL^−1^). Cells were evenly distributed to cryotubes (1.8 mL, Nunc, Thermo Fisher Scientific, Waltham, MA, USA) and pelleted by spinning at 1000 RPM for 3 min. Cells were maintained at 37 °C by placing vials into a solid block heater. The supernatant was discarded, reserving one-third volume of the cell pellet. Ischemia simulation was performed following the paraffin oil protocol developed by Vander Heide et al. [21] and Polewicz et al. [22]. Briefly, ischemia was simulated by adding a volume of paraffin oil equal to 3–4 mm depth (~500 μL) to the top of the cell pellet to prevent gas exchange from occurring. Samples were taken at 0, 30, 60 and 120 min of ischemia and at 5, 15- and 30-min reperfusion. The pH, PO_2_ and PCO_2_ were determined using i-STAT^®^ with cartridge CG8+ (Abbott Labs Corp., Macquarie Park, NSW, Australia).

### 2.4. Effect of Ischemia Reperfusion Injury on Fibroblast to Myofibroblast Differentiation

**Figure 1:**
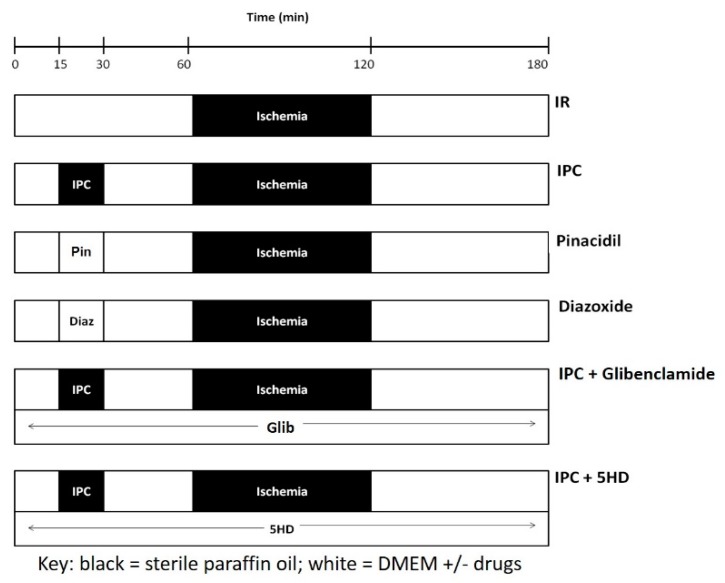
Protocols utilized to determine the effect of ischemia-reperfusion (IR) injury and of ischemic preconditioning (IPC) on rat cardiac fibroblast to myofibroblast differentiation. IR injury was produced by subjecting freshly isolated cardiac cells to ischemia at 37 °C. Reperfusion lasted 60 min. IPC was induced by preceding the IR with 15 min ischemia and 30 min reperfusion. IPC was mimicked by the application of K_ATP_ channel openers pinacidil (Pin, 3rd row) or diazoxide (Diaz, 4th row). The effect of blocking K_ATP_ current on IPC was tested by including glibenclamide (Glib, 5th row) or 5-hydroxydecanoate (5HD, 6th row). Key: black boxes represent periods where ischemia was mimicked by layering sterile paraffin oil onto the cells, whereas, white boxes represent periods where cells were covered with DMEM, with or without drugs, as specified.

Following dissociation, IR was simulated at 37 °C under sterile conditions. Dissociated cells were centrifuged and resuspended in DMEM. Reserving a small layer of supernatant, ischemia was simulated by the addition of paraffin oil and reperfusion was simulated by the removal of paraffin oil and the replacement with DMEM, as described above (Figure 1). Pelleted cells were exposed to 30, 60- or 120-min ischemia followed by 60 min reperfusion. Time controls (TC) were run, in which equal volumes of DMEM were applied in place of paraffin oil. Cells were then placed under culture conditions.

### 2.5. Effect of Ischemic Preconditioning on Ischemia Reperfusion Injury Induced Fibroblast to Myofibroblast Differentiation

Dissociated cells were centrifuged and resuspended in DMEM. Reserving a small layer of supernatant, IPC was studied by exposing pelleted cells to one episode of 15 min ischemia followed by 30 min reperfusion, prior to 60 min ischemia and 60 min reperfusion (Figure 1). Time controls were also run. Following IPC and IR, cells were placed under culture conditions. While the identities of all cell types within the cultures were not characterized in the current investigation, previous studies indicated that the unpassaged (P0) cultures contained ≥95% fibroblast purity [23,24]. Staining for factor VIII indicated that less than 1% of the culture were endothelial cells, while staining for desmin indicated that less than 1% of the culture were vascular smooth muscle cells [23,24].

### 2.6. Role of Adenosine Triphosphate-Sensitive Potassium Channels in Ischemic Preconditioning

Dissociated cells were centrifuged and resuspended in DMEM. Cells exposed to K_ATP_ blockers were subjected to 15 min IPC and 30 min reperfusion followed by 60 min ischemia and 60 min reperfusion (Figure 1). Glibenclamide (Glib, 10 μM, Sigma) or 5-hydroxydecanoate (5HD, 100 μM, Sigma) were administered 15 min prior to the onset of IPC, and maintained throughout the protocol. In other cells, IPC was mimicked by adding pinacidil (Pin, 50 μM, Sigma) or diazoxide (Diaz, 100 μM, Sigma) in place of the 15 min IPC, prior to 60 min ischemia and 60 min reperfusion. Cells were then placed under culture conditions.

### 2.7. Immunostaining for α-Smooth Muscle Actin

Once cultures were ~70–80% confluent, fibroblasts and myofibroblasts were fixed (2% paraformaldehyde, 30 min), permeabilized (1% triton X, 10 min) and blocked (1% BSA, 2 h). Fibroblasts and myofibroblasts were incubated overnight at 4 °C in monoclonal anti-α-SMA mouse IgG_2_ antibody (Vector labs, Burlingame, CA, USA), followed by 30 min in biotinylated horse anti-mouse IgG antibody (Vector labs, Burlingame, CA, USA). The primary antibody was diluted 1:5000 in NaCl/Pi buffer containing (mM): 140 NaCl, 2.7 KCl, 10 Na_2_HPO_4_, 1.8 KH_2_PO_4_ (adjusted to pH 7.3 using HCl). The secondary antibody was diluted 1:200 in NaCl/Pi buffer solution containing 1% BSA. Immunoreactivity was visualized using 3,3′-Diaminobenzidine (10 min) and cells were counterstained using Meyer’s hematoxylin. Antibody and staining specificity were determined by incubating cells with (a) no antibodies, (b) primary but no secondary antibody, and (c) secondary but no primary antibody. Non-specific staining was not observed in any of these controls (data not shown).

### 2.8. Data Analysis and Statistics

#### 2.8.1. Characterization of the Ischemic Conditions

Data are presented as mean percent change ± SEM (*n*). Statistical significance was determined using one-way ANOVAs with Scheffe posthoc tests where *p* values of <0.05 were considered significant.

#### 2.8.2. Ischemia Reperfusion Injury and Ischemic Preconditioning

**Figure 2:**
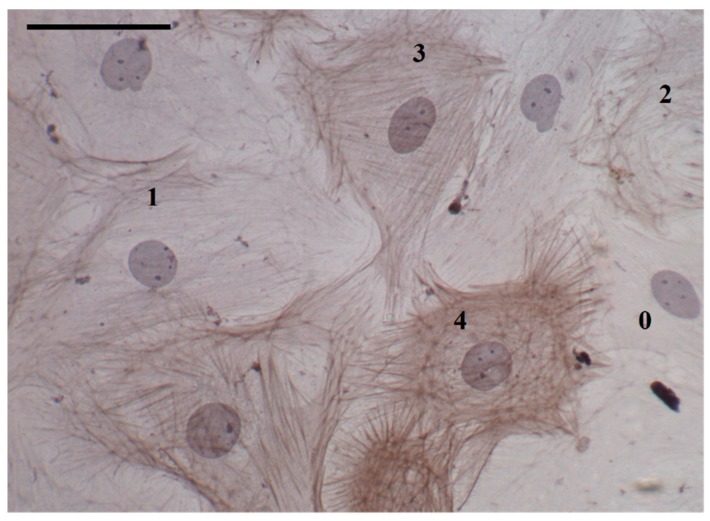
Cultured rat cardiac fibroblasts and myofibroblasts: characterization by α-smooth muscle actin (α-SMA) staining and stress fiber formation. Fibroblasts (0) were characterized by an absence of α-SMA staining, having no stress fibers. Both fully mature and immature myofibroblasts expressed α-SMA stress fibers, with the rating (1–4) reflecting the degree to which stress fibers filled the cell. Scale bar = 50 μm; magnification = 400×.

Slides were coded to guard against observer bias. Five representative images were taken from each slide and cells were assessed for the presence of α-SMA stress fibers. Cells were classified 0–4 depending on the extent of α-SMA expression (Figure 2). Undifferentiated fibroblasts did not express any α-SMA and were labelled 0. If the cell cytoplasm was occupied by greater than 75% α-SMA, the cell was classified as a fully mature myofibroblast and labelled 4. Cells with intermediate expression of α-SMA were classified as immature myofibroblasts and labelled ‘1–3′ if α-SMA expression was as follows: 1, less than 25%; 2, between 25% and 50%; and 3, between 50% and 75%.

For most analyses, the relative percentages of fibroblasts across treatments were compared, and no differentiation between immature and fully mature myofibroblast frequencies was made. Data are presented as mean percent (of the total number of cells analyzed within the representative images) ± SEM (*n*) unless specified otherwise. Statistical significance was determined using paired or unpaired Student’s t tests for single comparisons, and one-way or two-way ANOVAs with Scheffe posthoc tests for multiple comparisons. Significance was set at *p* < 0.05.

## 3. Results

### 3.1. Characteristics of Conditions During Ischemia and Reperfusion

**Figure 3:**
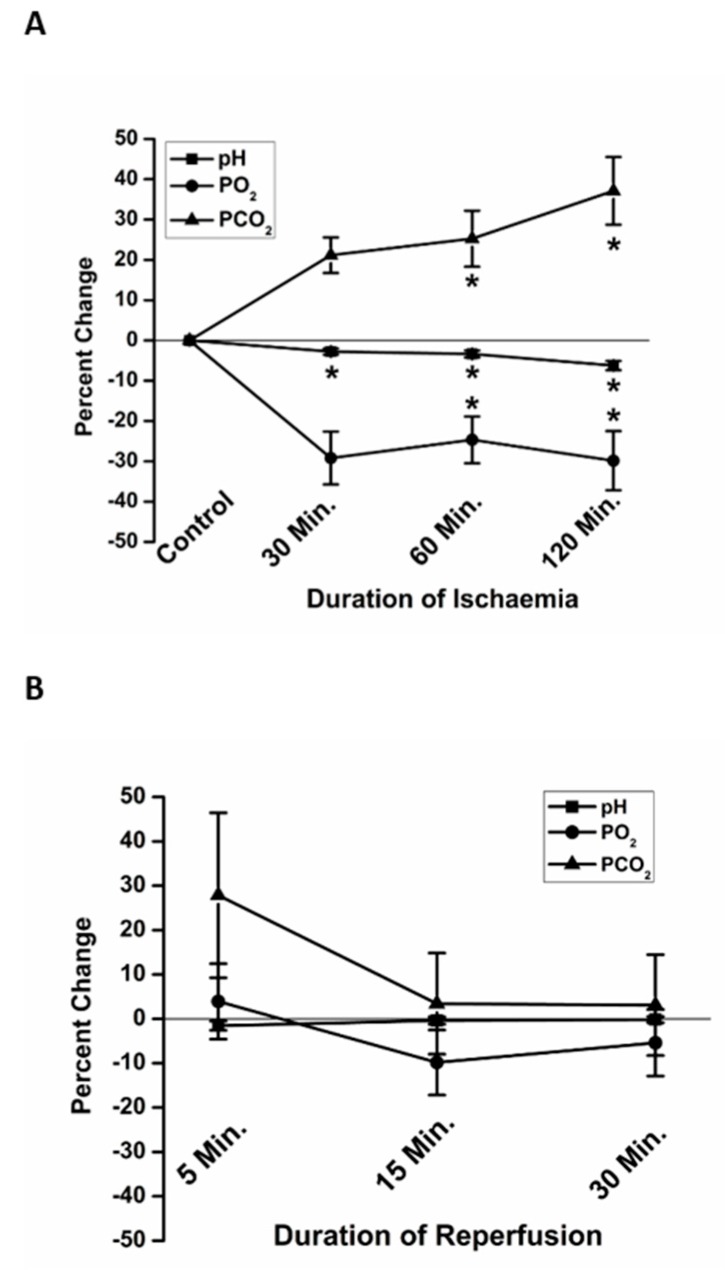
The in vitro changes in partial pressures of oxygen (PO_2_) and carbon dioxide (PCO_2_), and pH, mimicked changes known to occur in vivo during ischemia and reperfusion. At 60 and 120 min, significant acidosis (squares), elevation in PCO_2_ (triangles) and decline in PO_2_ (circles) were observed (**A**). All values returned to basal levels following reperfusion (**B**). Data are presented as percent change relative to Time 0, immediately prior to the beginning of ischemia. * *p* < 0.05 relative to Time 0.

To validate the in vitro use of paraffin oil as an effective protocol to simulate ischemia, we measured the pH, PO_2_ and PCO_2_ at 30, 60 and 120 min of ischemia (Figure 3A). The pH was significantly more acidic at 60 min (−3.37 ± 0.87% (7), percent change relative to the pre-ischemic baseline) and 120 min (−6.24 ± 1.13% (7)) of simulated ischemia, compared to Time 0. The PCO_2_ was significantly higher at 60 min (25.2 ± 6.9% (7)) and 120 min (37.1±8.4% (7)) of ischemia compared to Time 0. Compared to Time 0, PO_2_ was significantly lower at 30 min (−29.2 ± 6.6 (7)), 60 min (−24.7 ± 5.8% (7)) and 120 min (−29.8 ± 7.3% (7)) of ischemia.

The pH, PO_2_ and PCO_2_ were also measured at 5, 15- and 30-min reperfusion, following replacement of paraffin oil with fresh DMEM. Though there was variation in the rate of recovery, especially of PCO_2_ at 5 min, average pH, PO_2_ and PCO_2_ were not significantly different from the preischemic conditions at any of the times sampled in reperfusion (Figure 3B).

### 3.2. The Effects of Ischemia Reperfusion on Cardiac Fibroblast Differentiation

**Figure 4:**
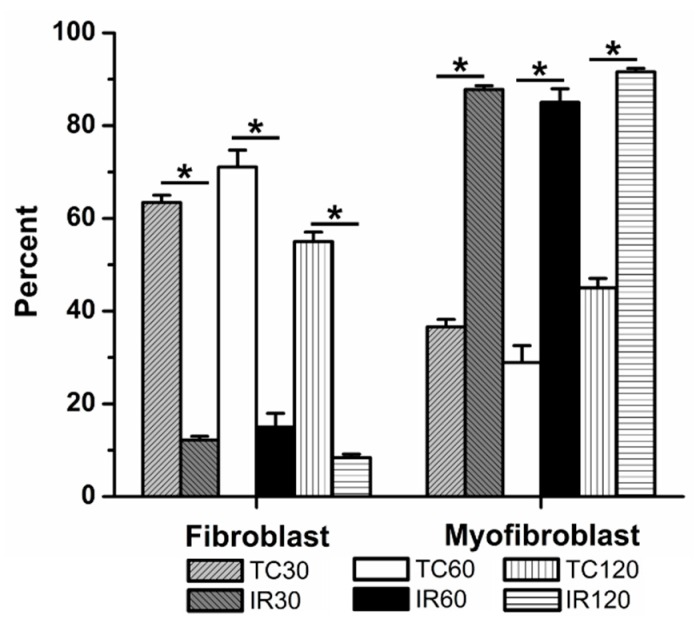
Ischemia reperfusion (IR) induced significant fibroblast to myofibroblast differentiation following 30, 60, and 120 min ischemia, relative to time controls (TCs). TCs are shown for 30, 60, and 120 min. The average percent of fibroblasts and myofibroblasts relative to the total number of cells analyzed are shown, ± SEM. * *p* < 0.05 between IR and TC for each time.

When exposed to an ischemic period of 30 min followed by 60 min reperfusion, significant fibroblast to myofibroblast differentiation was observed, when compared to time control (12 ± 1% (5) vs. 63 ± 2% fibroblasts (5), respectively, Figure 4). With 60 min and 120 min ischemia followed by 60 min reperfusion, significantly fewer fibroblasts were present than in their respective time controls. Following 60 min ischemia, 15 ± 3% (12) of the culture were fibroblasts, compared to 71 ± 4% (13) of cells in 60 min time controls. Similarly, after 120 min ischemia, only 8 ± 1% (5) of the population within the cultures was fibroblasts, compared to 55 ± 2% (4) in the 120 min time control.

### 3.3. The Effects of Ischemic Preconditioning on Cardiac Fibroblast Differentiation, and the Role of Adenosine Triphosphate-Sensitive Potassium Channels

**Figure 5:**
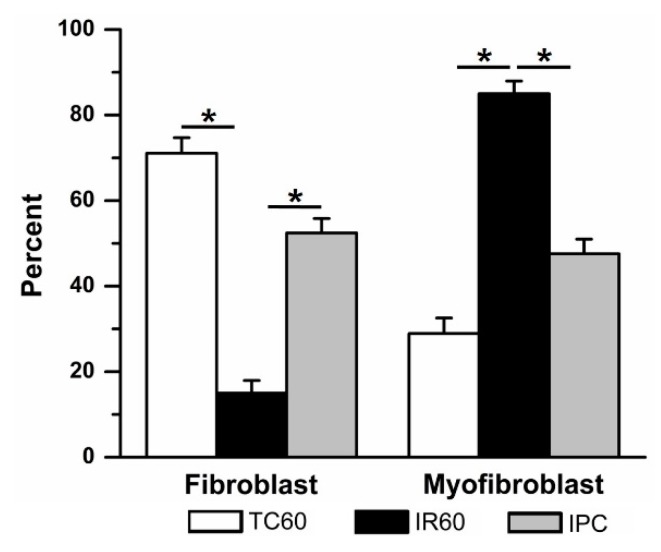
Ischemic preconditioning (IPC) prevented ischemia reperfusion (IR, 60 min)–induced fibroblast to myofibroblast differentiation. When compared to time control (TC), 60 min IR caused significant fibroblast to myofibroblast differentiation (data repeated from Figure 4). In contrast, adding a single episode of brief (15 min) ischemia and 30 min reperfusion prior to 60 min IR (IPC) significantly prevented fibroblast differentiation. * *p* < 0.05, comparisons indicated by the lines.

Ischemic preconditioning significantly attenuated IR-induced fibroblast differentiation (Figure 5). The IPC group had significantly more fibroblasts (52 ± 3%, (7)) compared to 60 min IR alone. These results suggest that while IR injury induced significant fibroblast-to-myofibroblast differentiation, IPC was able to protect against this effect. In time controls where cells were exposed to fresh DMEM in place of oil at each ischemic stage of the IPC/IR protocol, 72 ± 5% (4) of the cells were fibroblasts.

#### 3.3.1. The effects of Pinacidil and Diazoxide Treatment on Ischemia Reperfusion-Induced Cardiac Fibroblast Differentiation

Pinacidil has been shown to non-selectively activate K_ATP_ channels in myocytes [8,25] and reduce infarct size [26], indicating a role for K_ATP_ channels in IPC protection in cardiac myocytes. Mitochondrial K_ATP_ channels were also implicated in this process in cardiac myocytes, as application of the mK_ATP_ channel opener Diaz also mimicked the effects of IPC [27]. While K_ATP_ channels have been demonstrated in cardiac fibroblasts [28,29,30], it is not known if they play a similar role in IPC in cardiac fibroblasts. We investigated the effects of Pin and Diaz on IR injury in cardiac fibroblasts in vitro, to determine if these potassium channel openers could mimic the effects of IPC and significantly attenuate IR-induced fibroblast differentiation.

**Figure 6:**
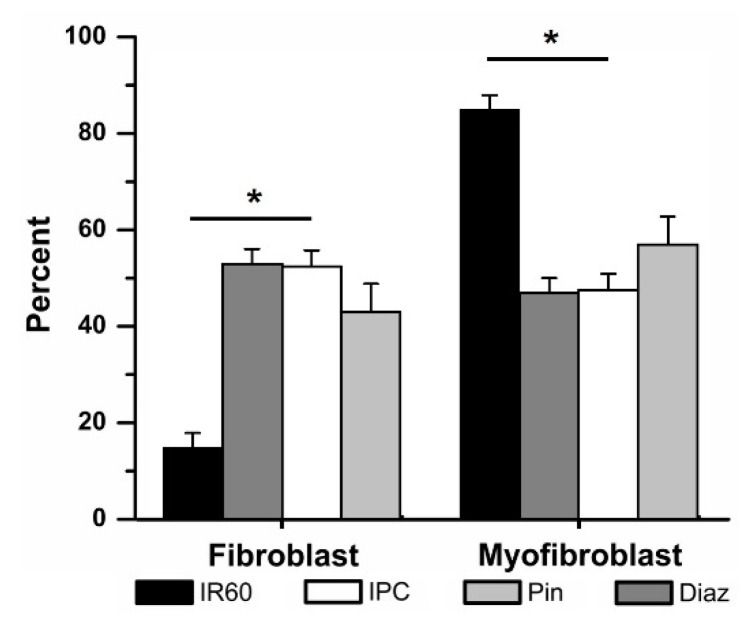
Ischemic preconditioning (IPC) could be mimicked by activating K_ATP_ channels with the non-specific opener pinacidil (Pin) and the mK_ATP_—selective opener diazoxide (Diaz). Replacing IPC with Pin (50 μM) or Diaz (100 μM) attenuated ischemia reperfusion (IR)-induced fibroblast to myofibroblast differentiation. * *p* < 0.05, IR vs. IPC and Diaz. The relative percentages of fibroblasts vs. myofibroblasts in IPC, Pin and Diaz were not significantly different.

Application of Pin (50 µM) in place of IPC prior to 60 min IR attenuated the differentiation of fibroblasts to myofibroblasts, compared to those cells exposed to 60 min IR alone (43 ± 6% (4) vs. 15 ± 3% (12) fibroblasts respectively, Figure 6) but did not cause a significant change. No significant differences were observed between the IPC and Pin-treated groups.

Similarly, Diaz significantly attenuated the IR-induced fibroblast to myofibroblast differentiation (Figure 6). Pretreatment with Diaz (100 µM) in place of IPC prior to 60 min IR, was associated with cultures containing 53 ± 3% (4) fibroblasts, significantly more than in the IR group. The percentage of fibroblasts within the Diaz-treated cells was not significantly different than that of the IPC groups. Neither drug affected fibroblast differentiation when tested in the time controls: when Pin was added to cells and subjected to the same protocol as the IPC cells, but without ischemia, 63 ± 9% (4) of the culture were fibroblasts, compared to 72 ± 5% (5) of cells in the IPC time controls. With Diaz present but no ischemia, 56 ± 13% (3) of the population were fibroblasts.

#### 3.3.2. The Effects of Glibenclamide and 5-Hdroxydecanoate on Cardiac Fibroblast Differentiation

The nonselective K_ATP_ channel blocker Glib [12] and the selective mK_ATP_ channel blocker 5HD [9,31] have been shown to reduce the effectiveness of IPC in cardiac myocytes. We used Glib and 5HD to determine if blockade of K_ATP_ channels affected IPC in cardiac fibroblasts, as is seen in cardiac myocytes.

**Figure 7:**
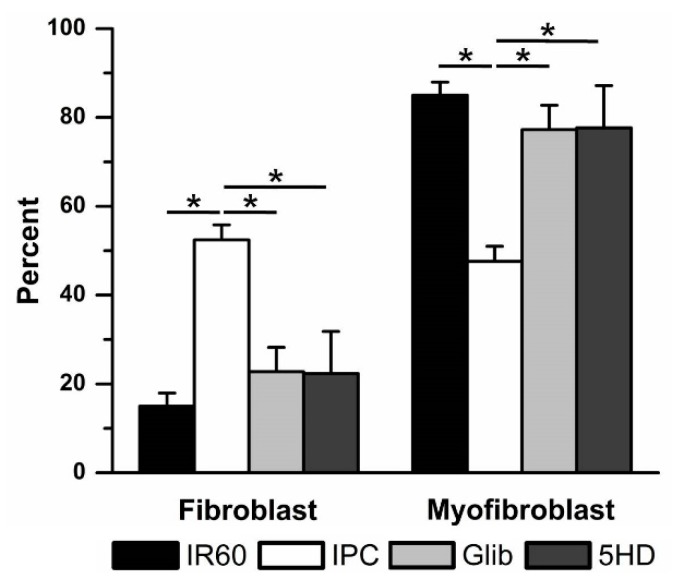
Ischemic preconditioning (IPC) was reduced by blocking K_ATP_ channels with the non-specific K_ATP_ blocker glibenclamide (Glib) and the mK_ATP_—selective K_ATP_ blocker 5-hydroxydecanoate (5HD). Blocking K_ATP_ channels with Glib (10 μM) or 5HD (100 μM) prevented IPC from reducing ischemia reperfusion (IR)-induced fibroblast to myofibroblast differentiation. * *p* < 0.05, comparisons indicated by the lines.

Both K_ATP_ blockers significantly reduced the effectiveness of IPC in preventing IR-induced fibroblast to myofibroblast differentiation (Figure 7). With 10 µM Glib present during IPC, the percentage of fibroblasts was reduced from 52 ± 3% (7, IPC) to 23 ± 5% (4, Glib). When cells were treated with 100 µM 5HD, only 22 ± 9% (3) of the culture were fibroblasts. There were no significant differences between the Glib- or 5HD- and IR-treated groups. Neither drug had any significant effect in the absence of ischemia. When used in time controls, Glib treatment was associated with 73 ± 2% (4) fibroblasts, while 5HD treatment was associated with 43 ± 15% (4) fibroblasts. These results were not significantly different from the IPC time control.

### 3.4. Effects of Ischemia Reperfusion Injury and Ischemic Preconditioning on Fibroblast Differentiation into Immature Vs. Fully Mature Myofibroblasts

Upon differentiation, myofibroblasts progress through a continuum of α-SMA expression as they mature into fully differentiated myofibroblasts with well-developed stress fibers [32,33,34]. Fibroblasts and fully differentiated myofibroblasts have distinct physiology, in the spectrum of signaling molecules they secrete and in the amount of collagen they are capable of producing. In addition, the amount of force a myofibroblast may produce during scar contracture is directly proportional to the extent of α-SMA expression [32,33,34]. Accordingly, cultures were scored as to the expression of α-SMA, to determine if IR injury and IPC influenced myofibroblast maturation as well as fibroblast differentiation.

**Figure 8:**
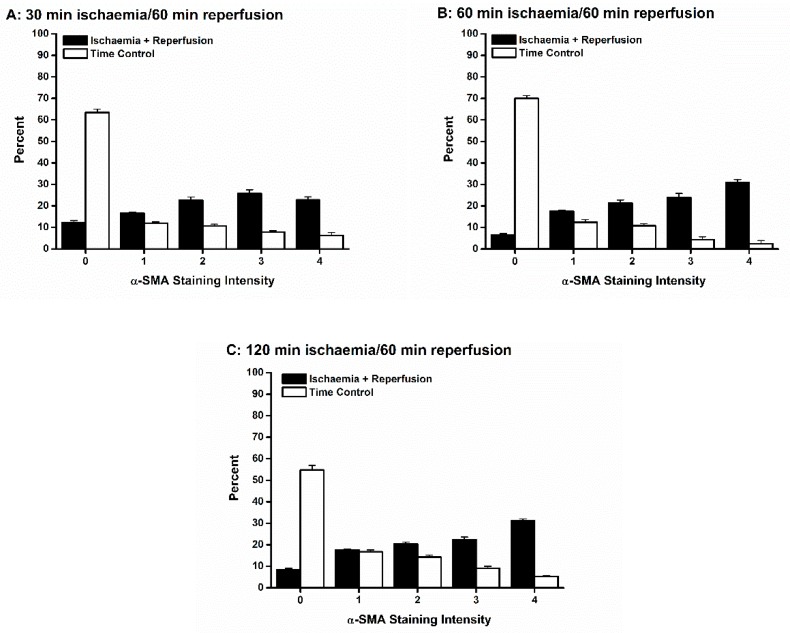
Ischemia reperfusion (IR) injury was associated with differentiation of fibroblasts into both immature and mature myofibroblasts. Each of the three durations of ischemia, 30 min (**A**), 60 min (**B**) and 120 min (**C**), yielded both immature (α smooth muscle actin (α-SMA) staining intensities 1–3) and mature (α-SMA staining intensity 4) myofibroblasts. In contrast, in each time control (TC), there were significantly more fibroblasts (α-SMA staining intensity 0), and of those fibroblasts that did differentiate, the resultant myofibroblasts tended to express less α-SMA stress fibers.

Ischemia of all durations followed by 60 min reperfusion was associated with differentiation of fibroblasts into a range of myofibroblasts with variable amounts of α-SMA-containing stress fibers (Figure 8). For all durations of ischemia, the trend was for the myofibroblasts to be highly differentiated. Only in the 30 min IR condition was the percent of fully differentiated myofibroblasts (staining category “4”) not larger than each of staining categories “1” through “3” (Figure 8A). In contrast, in all time controls, few myofibroblasts were present, and those tended to be less mature (Figure 8).

**Figure 9:**
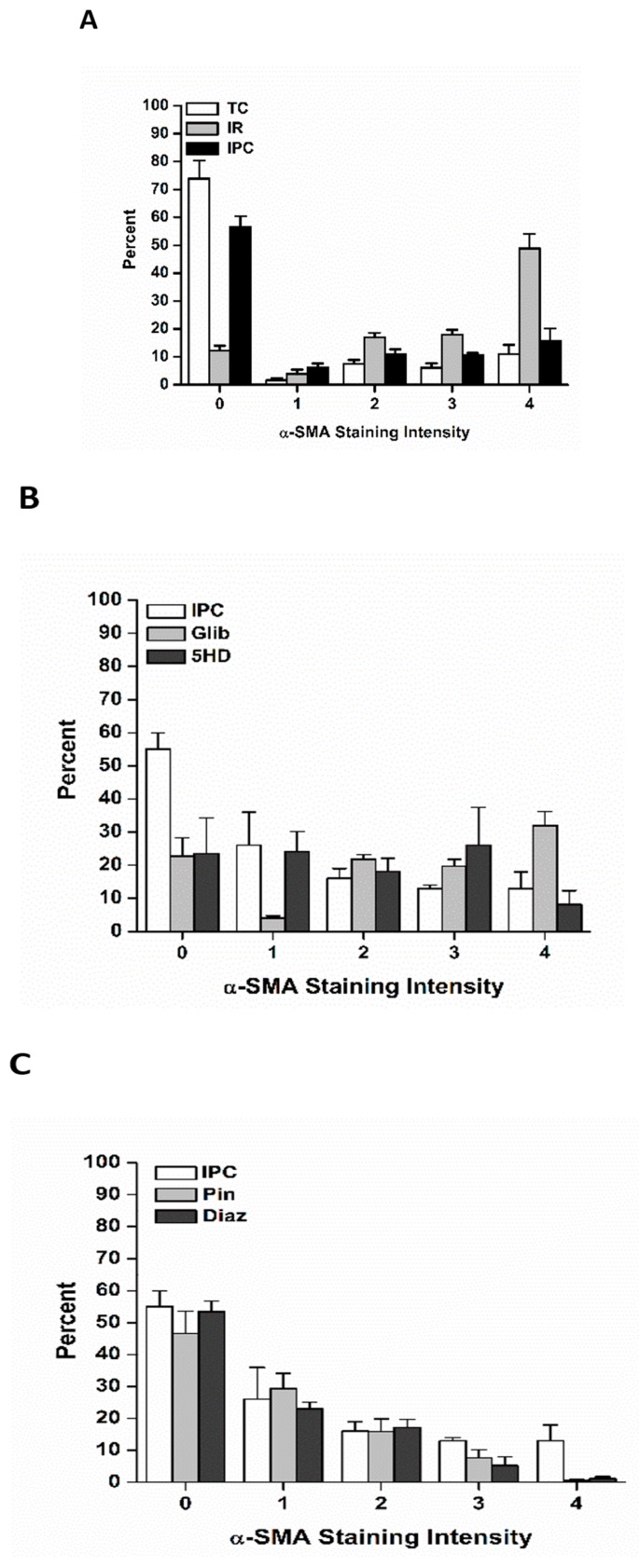
Ischemic preconditioning (IPC) influenced the relative proportions of immature vs. mature myofibroblasts. (**A)**: IPC (black bars) was associated with a significantly higher percentage of fibroblasts (α smooth muscle actin (α-SMA) staining intensity 0) and fewer fully mature myofibroblasts (α-SMA staining intensity 4) and a trend for immature myofibroblasts (α-SMA staining intensities 1–3). These trends were also observed when IPC was mimicked by opening K_ATP_ channels with pinacidil (Pin) (**B**) or diazoxide (Diaz) (**B**). In contrast, blocking K_ATP_ channels with either glibenclamide (Glib) (**C**) or 5-hydroxydecanoate (5HD) (**C**) was associated with fewer fibroblasts (α-SMA staining intensity 0) than following IPC (**B**,**C**).

Ischemic preconditioning shifted the relative percentages of myofibroblasts toward less mature myofibroblasts (Figure 9A), suggesting that not only were fewer fibroblasts differentiating, but maturation was delayed or prevented in the myofibroblasts that were produced. Application of Pin and Diaz had a similar effect (Figure 9B), while preventing IPC with Glib was associated with a greater proportion of more mature myofibroblasts (Figure 9C). A similar finding was observed when IPC was blocked by 5HD, though the trend toward mature myofibroblasts was not as pronounced as with Glib (Figure 9C).

## 4. Discussion

Cardiac fibroblasts are both functionally and phenotypically different from the classically studied cardiac myocytes [17,18,32]. The role of cardiac fibroblasts and myofibroblasts in the development of fibrosis following myocardial ischemia makes them potential candidates to unravel the pathophysiology of ischemic heart disease. Until now, the effects and mechanisms of IR injury and IPC on cardiac fibroblast to myofibroblast differentiation have not been studied. We modified the paraffin oil method of simulating IR injury in order to investigate the role of IR injury, IPC and K_ATP_ channels in adult rat ventricular cardiac fibroblasts.

### 4.1. Characterization of the Ischemic Conditions

To validate the use of paraffin oil as an effective method of simulating ischemia in vitro, we measured the pH, PO_2_ and PCO_2_ at 30, 60 and 120 min of ischemia, and the recovery of these parameters to pre-ischemic levels at 5, 15- and 30-min reperfusion. Our results demonstrate that the application of paraffin oil resulted in a significant increase in the PCO_2_, decrease in the PO_2_ and acidosis, all of which recovered rapidly following removal of the paraffin oil (Figure 3). In vivo studies have demonstrated that myocardial ischemia results in increased PCO_2_, decreased PO_2_ [35] and acidosis [36]. Our results are consistent with ischemic conditions in vivo and suggest that paraffin oil is a valid method of simulating ischemic conditions in vitro. However, not all elements of ischemia and reperfusion in vivo can be mimicked by this in vitro assay. The in vivo tissue architecture and gap junctional communication are lost in the dissociation process. Reperfusion in vivo is associated with activation of neutrophils and free oxygen radical production by neutrophils and endothelial cells [37]; in vitro assays may lack these elements.

### 4.2. Ischemia Reperfusion Injury and Ischemic Preconditioning in Cardiac Fibroblasts

To the best of our knowledge, this is the first study to demonstrate that cardiac fibroblasts differentiate into myofibroblasts in response to IR injury. This is also the first study to demonstrate that IPC ameliorates the IR injury-induced differentiation of cardiac fibroblasts into myofibroblasts. Few other studies have examined cardiac fibroblasts following ischaemia and reperfusion, or hypoxia and reoxygenation. Vivar et al. [38] studied IR injury-induced death and the protective effect of insulin-like growth factor 1 in cultured neonatal rat cardiac fibroblasts. Zhou et al. [39,40] established a model of cultured rat neonatal and adult cardiac fibroblasts, in which they have considered the deleterious effects of hypoxia and reperfusion, comparing biochemical and morphological changes in cultured fibroblasts to those of cultured ventricular myocytes [39]. They also found that conditioned media from cultured cardiac fibroblasts subjected to hypoxia and reoxygenation did not protect cardiac myocytes from IR damage, while factors from other non-myocytes of mesenchymal origin did [40]; however, these authors did not consider IR injury beyond changes associated with fibroblast death, or IPC, in their model. Lefort et al. [41] reported that cultured human ventricular fibroblasts produced secretomes in response to 5 h of hypoxia and 24 h reoxygenation, which reduced cardiac myocyte death during the hypoxia/ reoxygenation challenge. These authors also reported that stimulation of the metabotropic purine P2Y11 receptor in cultured human ventricular fibroblasts at the onset of reoxygenation reduced fibroblast to myofibroblast differentiation [41], suggesting that Gq and Gs protein-coupled pathways modulate fibroblast differentiation in response to hypoxia and reoxygenation.

Ischemic preconditioning may be an effective therapeutic strategy not only to protect the heart against myocyte injury and death, but also to prevent the development of inflammation and fibrosis following myocardial infarction. Hypoxia/reoxygenation-induced secretome release from cultured human ventricular fibroblasts was associated with a pro-inflammatory response; this effect was diminished if P2Y11 receptors were activated during reoxygenation [41]. P2Y11 receptor activation also reduced fibroblast to myofibroblast differentiation in this model [42], similar to our findings (Figure 5, Figure 6, Figure 7, Figure 8 and Figure 9). This may be significant when considering the degree of fibrosis associated with IR injury.

Fibrosis is also strongly correlated with fibroblast to myofibroblast differentiation [31,32] and contributes to the pathogenesis of arrhythmias and heart failure [17]. Furthermore, fibroblasts have been shown to protect the myocardium via the production of ‘currently undefined’ substances [43]. Therefore, preserving fibroblasts in the infarct zone following an ischemic insult will not only help to reduce the number of myofibroblasts in the heart, limiting reactive fibrosis [17], it may also help to protect the myocardium against further IR injury by other mechanisms. In addition, specific cardiac fibroblast G-protein-coupled receptor kinase 2 (GRK2) knockout in mice was shown to reduce infarct size, degree of fibrosis and inflammation following IR injury [44]. These results suggests that upregulation of GRK2 contributes to fibrosis, inflammation, and myocyte death through fibroblast-specific actions. It would therefore be interesting to determine the effect of IPC on GRK2 activation in cardiac fibroblasts.

### 4.3. The Role of Adenosine Triphosphate-Sensitive Potassium Current in Preventing Fibroblast to Myofibroblast Differentiation

The activation of K_ATP_ channels occurs during IPC in myocytes and has been shown to protect against IR injury [28,29,30]. Adenosine triphosphate-sensitive K channels have recently been described in cardiac fibroblasts [28,29,30].

We investigated whether K_ATP_ channels may mediate the protective effect of IPC in cardiac fibroblasts. Our results demonstrated for the first time that the opening of pK_ATP_ and mK_ATP_ channels with Pin and selectively opening mK_ATP_ channels with Diaz mimicked the effects of IPC and attenuated IR-induced fibroblast differentiation (Figure 6). Furthermore, our results indicate that inhibition of pK_ATP_ and mK_ATP_ channels with Glib and selective inhibition of mK_ATP_ channels with 5HD abolished IPC-induced protection against fibroblast differentiation. These results suggest that mK_ATP_ channels are activated during IPC to prevent fibroblasts from differentiating into myofibroblasts.

#### Involvement of Mitochondrial Vs. Sarcolemmal Adenosine Triphosphate-Sensitive Potassium Channels in Ischemic Preconditioning in Fibroblasts

Activation of sK_ATP_ channels in cardiac myocytes causes sarcolemmal hyperpolarization at rest [8]. This current also enhances membrane repolarization, shortening the action potential duration and limiting voltage-gated Ca^2+^ current [12]. This action leads to reduced Ca^2+^ entry into cardiac myocytes during ischemia [13].

Cardiac fibroblasts do not appear to be excitable cells [19,20,45,46,47]. While immunohistochemical [48] and pharmacological studies [48,49,50] have suggested that L-type Ca^2+^ channels may play a role in cardiac fibroblasts and myofibroblasts, none of the electrophysiological patch clamp studies on cardiac fibroblasts or myofibroblasts have provided evidence of voltage-gated Ca^2+^ currents [19,20,28,29,30,45,46,47,51,52,53,54,55,56,57,58,59]. In the absence of voltage-gated Ca^2+^ currents, hyperpolarization due to pK_ATP_ current would not be predicted to reduce Ca^2+^ entry through the plasmalemma, as it would for myocytes. Conversely, as non-selective cation conductances appear to be present [48,54], hyperpolarization would be predicted to increase Ca^2+^ entry through these channels. Thus, we hypothesized that cardioprotection from the activation of K_ATP_ channels in cardiac fibroblasts, arises primarily from the activation of mK_ATP_ channels. In fibroblast as well as myocyte mitochondria, activation of mK_ATP_ current is predicted to reduce Ca^2+^ current through the Ca^2+^ uniporter and prevent Ca^2+^ overload [10,11]. Hence, as K_ATP_ channels appear to be an end-effector of signaling in IPC, it is reasonable to hypothesize that the mitochondrial channel is preferentially activated in cardiac fibroblasts. This possibility is further supported by evidence that Wistar rat left-ventricular fibroblasts do not strongly express pK_ATP_ currents [30]. Intriguingly, Benamer et al. [30] found that K_ATP_ currents were larger in fibroblasts isolated from infarction scars and from the border zone than in fibroblasts from the non-infarcted regions of the infarcted hearts, or from non-infarcted control hearts. These authors did not determine if the fibroblasts may have differentiated into myofibroblasts in the infarction and border zones. If so, this suggests that calcium entry into myofibroblasts may be enhanced due to pK_ATP_ current, in the infarction scar and border zone. The significance of such calcium entry is not currently known.

Our results demonstrated that activating mK_ATP_ channels mimicked the effects of IPC by reducing fibroblast differentiation, while blocking mK_ATP_ channels attenuated the effects of IPC and resulted in increased fibroblast differentiation (Figure 7). The magnitude of selective activation of mK_ATP_ channels with Diaz was not significantly different from non-selective activation with Pin, suggesting that the primary effect was mitochondrial. Similarly, non-selective blockade with Glib had the same effect as selectively blocking mK_ATP_ channels with 5HD, suggesting that the primary effect was inhibition of the mitochondrial channel. These results support our hypothesis that mK_ATP_ channels are important in the development of IPC-mediated protection against IR-induced fibroblast differentiation.

### 4.4. Immature vs. Fully Mature Myofibroblasts

Myofibroblasts vary in the amount of α-SMA they express, and the corresponding degree of mature stress fibers formed in these contractile cells [52]. In addition, when compared to fibroblasts, myofibroblasts secrete a distinct range of signaling molecules, and are much more rapidly capable of remodeling the extracellular matrix (ECM) [17,32,34]. As myofibroblasts may be associated with maladaptive fibrosis in the heart [17], the degree to which the myofibroblasts mature following IR injury is of clinical interest.

We found that IR injury was associated with more mature myofibroblasts, and that IPC tended to prevent or delay myofibroblast maturation (Figure 8 and Figure 9). In addition, modulation of K_ATP_ channel recruitment influenced myofibroblast maturation: treatment with Pin and Diaz tended to prevent or delay maturation, while Glib and 5HD tended to promote it following IR injury. These results suggest that IPC may be important not only in reducing the total number of myofibroblasts being generated, but also in delaying or preventing maturation. Both effects would be predicted to reduce fibrosis in the wounded heart, and so, to reduce mechanical dysfunction and the risk of arrhythmias.

While to our knowledge, this is the only study of IR injury and IPC in cardiac fibroblasts, the effects of persistent hypoxia and ischemia in skin wound healing have been investigated. In a rat model of sustained hind limb ischemia, a wound on the ischemic foot failed to heal as well as a matching wound on the non-ischemic control foot [60]. This was attributed to delayed production and maturation of myofibroblasts within the granulation tissue, preventing wound closure and contracture [60]. Hypoxia was found to have a similar effect [61,62]. These studies lead to the hypothesis that during ischemia, cardiac fibroblasts may not as readily differentiate into myofibroblasts, and that it is during reperfusion that the differentiation is triggered in the absence of K_ATP_ channel activation by IPC. This is in agreement with the hypothesis that the mitochondrial isoform of this channel is the primary one at work in the fibroblast, as it is during reperfusion that protection of the inner mitochondrial membrane potential is important to prevent Ca^2+^ overload [10,11]. Like myocytes, cardiac myofibroblasts are believed to express the Na^+^/Ca^2+^ exchanger [48]. The Na^+^/H^+^ exchanger NHE1 has been demonstrated in cardiac fibroblasts [63]. Only in reperfusion is there a pH gradient which affects the Na^+^/H^+^ and Na^+^/Ca^2+^ exchangers, increasing cytosolic and subsequently, mitochondrial Ca^2+^ levels [10]. Hence, it is during reperfusion that activation of mK_ATP_ channels is likely to have a protective effect in preventing fibroblast differentiation. The role of membrane potential in fibroblast to myofibroblast differentiation has not been studied; however, modulation of membrane potential is known to affect migration [24,48], contraction and proliferation [19,48] of myofibroblasts.

### 4.5. Important Considerations

One caveat of research concerning mK_ATP_ channels is that the existence of these channels has not been conclusively demonstrated (reviewed in [8]). While currents from these channels have been recorded in rat liver mitochondrial inner membrane [7], these authors could not be absolutely certain that their mitochondrial preparations were free of contamination by the plasma membrane. Other indirect studies of mitochondrial function have provided supportive evidence of this channel’s existence and that it plays a role in protecting the mitochondria from Ca^2+^ overload during IR injury, but the molecular identity of this channel is still unknown (reviewed in [8]). In our studies, we have been careful to apply the selective mK_ATP_ blockers and activators at appropriate concentrations [25,64] to avoid non-selective effects. At present, the technological constraints of recording K_ATP_ currents from the inner membranes of individual mitochondria isolated from cardiac fibroblasts prevent us from providing direct evidence that IPC and the selective mK_ATP_ drugs modulated mK_ATP_ channels in these cells, as we suggest. Hence, the role and nature of K_ATP_ channels within the mitochondria during IPC in cardiac fibroblasts are to be treated with due caution, as they are in cardiac myocytes [8].

Isoflurane is known for its ability to open K_ATP_ channels and exert cardioprotective effects on the heart [65,66]. Our animals were only briefly (<10 min) exposed to isoflurane during the isolation procedure. Isoflurane-induced IPC was not observed after 15 min exposure in mice [66], suggesting that the duration of exposure in our studies was unlikely to evoke IPC. Our results also suggest that our cardiac fibroblasts were not under the protection of isoflurane IPC, as IR injury caused significantly more fibroblast differentiation than IPC or matched time controls. It would be interesting, however, to investigate the effects of isoflurane and other volatile gases on IR injury and cardiac fibroblast differentiation.

## 5. Conclusions

Using an in vitro model of IR injury, we demonstrated that cardiac fibroblasts differentiate into myofibroblasts in response to IR-injury. Furthermore, we showed that IPC reduced the amount of IR-induced differentiation. We are the first to demonstrate that the activation of K_ATP_ currents mimic the protection of IPC and protect against IR-induced fibroblast to myofibroblast differentiation, and that blockade of these channels reduces the effectiveness of IPC. While we are aware that the molecular identity of mK_ATP_ is not yet known [8], we believe that the electrophysiological evidence [7] supports the existence of this channel. Our data therefore suggest that IPC is mediated by specific activation of the mK_ATP_ in cardiac fibroblasts. This research will hopefully help in future development of new therapeutic treatments for post-myocardial infarction fibrosis.
